# 

**Published:** 1864-02

**Authors:** 


### BOOKS AND PAMPHLETS RECEIVED.

*A Treatise on Pharmacy.* By Edward Parish. Philadelphia . Blanchard & Lea; 1864.

*The Medical Formulary.* By Benjamin Ellis, M. D. Philadelphia: Blanchard & Lea; 1864.

*The Nervous and Vascular Connection between the Mother and Fœtus in Utero.* By John O'Reilly, M. D., F. R. C. S. I. New York: Robert Craighead; 1864.

*Eleventh Annual Meeting for years* 1861, '2 *and* '3 *of the Illinois State Medical Society, held at Jacksonville May* 5, 1863. Chicago: Geo. H. Fergus; 1863.

*Tenth Annual Report of the Trustees of the State Lunatic Hospital at Taunton, October,* 1863. Boston: Wright & Potter; 1864.

*Death: Its Economy and Beneficence. An Address delivered before the Medical Class of the University of Vermont, Tuesday evening, June 9th,* 1863, by Henry M. Seely, M. D. Burlington; 1863.

*The American Journal of Insanity, edited by the Medical Officers of the New York State Lunatic Asylum. January,* 1864. Utica, N. Y: Curtiss & White; 1864.

